# Arterial spin labeling perfusion MRI differentiates between radiation necrosis and tumor in brain metastases treated with stereotactic radiosurgery

**DOI:** 10.1093/noajnl/vdaf091

**Published:** 2025-05-07

**Authors:** Henry H Nguyen, Nathan N Ng, Shriya Awasthi, Hannes Vogel, Michael Iv

**Affiliations:** Division of Neuroimaging and Neurointervention, Department of Radiology, Stanford University School of Medicine, Stanford, California, USA; Division of Neuroimaging and Neurointervention, Department of Radiology, Stanford University School of Medicine, Stanford, California, USA; Division of Neuroimaging and Neurointervention, Department of Radiology, Stanford University School of Medicine, Stanford, California, USA; Division of Neuropathology, Department of Pathology, Stanford University School of Medicine, Stanford, California, USA; Division of Neuroimaging and Neurointervention, Department of Radiology, Stanford University School of Medicine, Stanford, California, USA

**Keywords:** arterial spin labeling perfusion MRI, brain metastases, cerebral blood flow, radiation necrosis, stereotactic radiosurgery

## Abstract

**Background:**

Accurate differentiation between radiation necrosis (RN) and tumor in brain metastases (BM) treated with stereotactic radiosurgery (SRS) can be challenging, but it is important because an accurate diagnosis impacts clinical management. In this study, we evaluated the utility of arterial spin labeling perfusion MRI (ASL-MRI) to accomplish this task.

**Methods:**

We retrospectively evaluated 45 patients with 52 previously irradiated BM who had ASL-MRI prior to surgical resection. Histopathology served as the ground truth diagnosis of tumor and RN. Maximum cerebral blood flow (CBF) values were obtained within the contrast-enhancing lesions of interest and the pons (for normalization) on quantitative ASL-MRI CBF maps. In a subgroup analysis, patients with both pre-SRS and post-SRS ASL-MRIs were included, and CBF values were obtained at both timepoints.

**Results:**

Compared with RN, tumor had increased mean absolute and normalized CBF (*P* < .0001). In the subgroup analysis of patients with pre-SRS and post-SRS ASL-MRIs, change in absolute CBF (∆CBF) and normalized CBF (∆nCBF) of tumor showed higher absolute and percent differences between both timepoints (*P* < .02). Performance of ∆CBF and ∆nCBF (area under the receiver operating characteristic curve [AUROC] 0.80–0.89) acquired from 2 ASL-MRIs was comparable but not superior to CBF and nCBF (AUROC 0.90) acquired from single timepoint post-SRS ASL-MRI.

**Conclusions:**

Increased CBF, whether absolute or normalized, on post-SRS ASL-MRI performed well to differentiate tumor from RN in BMs treated with SRS. Addition of pre-SRS CBF measurements did not improve the performance. ASL-MRI is a promising imaging tool to distinguish RN from tumor in this patient population.

**Key Points**

- ASL-CBF differentiates between tumor and radiation necrosis in post-SRS brain metastases.
- ∆CBF from pre- and post-SRS ASL-MRIs performs comparably and is not superior to CBF from single post-SRS ASL-MRI.
- CBF of RN is independent of time after radiotherapy.

**Importance of the Study**

Stereotactic radiosurgery (SRS) has revolutionized the treatment of brain metastases. However, differentiating between radiation necrosis (RN) and tumor progression following SRS is difficult due to the limited ability of conventional gadolinium-enhanced MRI to effectively distinguish between these conditions. Therefore, more effective diagnostic imaging tools are needed because incorrect diagnoses can lead to suboptimal treatment-related decision-making. In this study, we investigated arterial spin labeling perfusion MRI (ASL-MRI) as a noninvasive imaging tool to differentiate between RN and tumor in this patient population. Our findings show that blood flow associated with RN is significantly lower than tumor and is independent of time after radiotherapy. ASL-MRI is, therefore, a promising and widely available tool to inform clinical management in these patients.

Neuroimaging plays a vital role in determining the efficacy of stereotactic radiosurgery (SRS) in patients with brain metastases (BM). Accurate differentiation between residual or recurrent tumor and radiation necrosis (RN) is crucial in determining whether patients demonstrate a beneficial or suboptimal response to SRS.^1,2^

Conventional gadolinium-enhanced MRI cannot consistently and reliably differentiate between these 2 conditions.^3–7^ The challenge lies in the overlap of imaging appearance, particularly on postcontrast images.^3,8^ Kuo et al. found that delta T1 as a marker of contrast-enhancing volume performed poorly (AUROC 0.64) to differentiate between tumor and RN in patients with BM previously treated with SRS.^5^ Solely relying on contrast enhancement, which is a nonspecific marker of blood-brain barrier disruption, can therefore lead to incorrect diagnoses in this patient population.^9,10^

Advanced imaging techniques, such as perfusion MRI, which highlight physiological differences between tumor and RN, have shown to be promising.^1,5,9,11–13^ Tumor physiology is characterized by a drive for continuous growth and proliferation, which is a process that demands increased nutrient-rich resources. The increased demand for nutrients in turn results in an increased need for blood flow, which can be detected and measured by perfusion MRI.^5,14^ In contrast, the drive for continuous growth and proliferation is not a classic feature of RN, which explains why it is often characterized by reduced blood flow.^5,9,15^ Studies have shown the value and good performance of perfusion MRI metrics such as cerebral blood flow (CBF) derived from arterial spin labeling (ASL) and relative cerebral blood volume and fractional tumor burden derived from dynamic susceptibility contrast (DSC) MRI to differentiate between tumor and RN^5,6,9,11–13,16–18^ and to predict clinical outcomes.^10,13,19–22^ ASL may be more helpful than DSC in this setting as posttreatment BM can show hemorrhage, which can produce susceptibility artifacts on DSC that render postprocessed images uninterpretable.^10^

In this study, we evaluated the utility of ASL-MRI to differentiate between tumor and RN in patients with BM previously treated with SRS. Given previously published studies and our own clinical experience, we hypothesized that ASL-MRI would perform well to differentiate between these 2 conditions. In a subgroup of patients with pre-SRS and post-SRS ASL-MRIs, we determined if the addition of baseline pre-SRS CBF improved ASL-MRI’s performance to distinguish between tumor and RN. Finally, we investigated whether time after radiotherapy affects ASL-MRI’s performance, as RN is known to typically occur within 2 years after radiotherapy.^8^

## Materials and Methods

### Patients

This retrospective study was approved by our institutional review board. Through an electronic neuropathology database, we obtained a list of patients with BM previously treated with SRS who underwent surgical resection and histopathological evaluation between January 2010 and December 2023.

Inclusion criteria were as follows: 18 years of age or older, history of BM previously treated with SRS, availability of post-SRS ASL-MRI showing at least one suspicious contrast-enhancing lesion (defined by contrast-enhancing growth post-SRS with the longest lesion diameter measuring ≥10 mm), surgical resection occurring within 60 days of the post-SRS ASL-MRI, and a documented diagnosis of either tumor or RN following histopathological evaluation of the resected lesion. Patients were excluded if at least one inclusion criterion was not met.

In a subgroup analysis, ASL-MRIs of patients with available pre-SRS and post-SRS (prior to surgical resection) imaging were evaluated to determine absolute and percent differences in CBF (∆CBF, defined as post-SRS CBF—pre-SRS CBF) and normalized CBF (∆nCBF, defined as post-SRS nCBF—pre-SRS nCBF) between the 2 timepoints.

Clinical demographics, histopathologic information, and treatment history were obtained through the electronic health record.

### Image Acquisition and Postprocessing

MRIs were performed on either 1.5T or 3T scanners (GE Healthcare) and were acquired as part of the brain metastasis protocol of our institution, which included pre-gadolinium 3D T1-weighted, ASL, and post-gadolinium 3D T1-weighted images.

ASL imaging was performed with a 3D background-suppressed fast spin-echo technique without vascular suppression using a pseudocontinuous labeling time of 1.5 seconds, followed by a 2-second postlabeling delay. Imaging parameters included TR/TE = 4000/10 ms, in-plane spatial resolution = 3 mm, section thickness = 4 mm, skip = 0 mm, with the labeling plane at the level of the foramen magnum. Postprocessing was performed by an automated reconstruction script on the scanner that sent quantitative CBF images directly to PACS.

### Image Analysis

A third-year medical student (H.H.N.) performed all image segmentation using PACS. Post-gadolinium images of target lesions were spatially localized to the quantitative CBF images using the cross-reference tool. Three regions of interest (ROIs) were placed around each target lesion on separate image slices with the highest signal based on visual assessment (“hot spot” analysis), and the average of the maximum CBF values acquired from these 3 ROIs was used to obtain a single absolute CBF value for each lesion. Another three 50 mm^2^ ROIs were then placed in the pons of the same patient, and their maximum CBF values were averaged to create a single CBF value for normalization. The pons was chosen for normalization because its metabolism and volume are least affected by disease.^23^ All ROIs were reviewed, confirmed, and adjusted as necessary by a board-certified neuroradiologist with over 10 years of experience in neuro-oncology imaging (M.I.). Each lesion was analyzed separately in patients with more than one lesion. For patients with additional pre-SRS ASL-MRI, the same technique was performed to obtain CBF values of the lesions of interest. All image analyses were performed in a blinded fashion without knowledge of the final histopathological diagnosis.

### Ground Truth Diagnosis

The diagnosis of tumor or RN was determined by histopathological evaluation (and obtained from the electronic health record) following surgical resection of a patient’s irradiated brain metastasis. Lesions were classified as RN if there was no mention of tumor present in the pathology reports. Lesions were classified as tumor if there was no mention of RN or reactive changes in the pathology reports. Descriptions of lesions that had any combination of the 2 were classified as mixed regardless of the percentage of RN or tumor present as this was not consistently stated in all cases.

### Statistical Analysis

Descriptive statistics were used to report demographics, clinical information, and CBF metrics. The Mann–Whitney *U* test was used to compare CBF metrics between tumor and RN. The performance of CBF metrics to distinguish between tumor and RN was evaluated with area under the receiver operating characteristic curve (AUROC). The Youden index was calculated to obtain the optimal threshold values, which were then used to determine the sensitivity and specificity based on the ROC curves. In these primary analyses, we evaluated data from lesions that were definitively tumor or RN and excluded mixed lesions, as the proportion of tumor versus RN in those samples was not consistently known. However, mixed lesions were included in supplementary analyses, and CBF metrics of mixed lesions were compared with those of RN and tumor using one-way ANOVA with Tukey’s multiple comparison tests with multiplicity-adjusted *P* values.

To determine the relationship of CBF and time elapsed since radiotherapy in RN, analyses of RN-only lesions were performed. First, RN lesions were stratified based on 2 time windows following SRS (5 months to 2 years and >2 years). This stratification was based on knowledge that most cases of RN occur within 6 months to 2 years following SRS.^8^ A Mann–Whitney *U* test was conducted using the 2 time windows. In addition, a simple linear regression, with time elapsed since radiotherapy represented as a continuous independent variable and CBF as the dependent variable, was performed.

For all analyses, *P* < .05 was considered statistically significant. GraphPad Prism software (version 10.1.2) was used to conduct all statistical analyses and generate illustrative figures.

## Results

### Patient Demographics

A total of 651 patients were screened, and a total of 45 patients (mean age: 56.8, SD 10.7, range 25–78, 30 females) with 52 suspected metastases (31 tumor, 21 RN) were included in the final cohort. Twelve patients were excluded due to their young age (<18 years), 579 patients were excluded due to the absence of available post-SRS ASL-MRI, and 4 patients were excluded for undergoing surgical resection more than 60 days after post-SRS ASL-MRI. Eleven patients were excluded from the primary analyses though included in supplementary analyses, because the pathology reports of resected tissue showed mixed lesions. The primary cancers of origin were as follows: lung (*n* = 20), melanoma (*n* = 12), breast (*n* = 8), colorectal (*n* = 2), renal (*n* = 2), ovarian (*n* = 2), testicular (*n* = 2), sarcoma (*n* = 1), endometrial (*n* = 1), thymic (*n* = 1), and cardiac (*n* = 1). Median interval time between initial SRS treatment and post-SRS ASL-MRI was 310 days (SD 704.7, range: 8–3547). Median interval time between post-SRS ASL-MRI and histopathological evaluation was 11 days (SD 13.2, range: 1–52). All lesions received CyberKnife as the SRS treatment at a median dose of 24 Gy (SD 6.9, range 18–49) in a median fraction of 1 (SD 1.2, range 1–6). At the time of post-SRS ASL-MRI acquisition, 9 patients with 12 lesions had received both chemotherapy and immunotherapy, 26 patients with 29 lesions had received only chemotherapy, 6 patients with 7 lesions had received only immunotherapy, and 4 patients with 4 lesions had received neither chemotherapy nor immunotherapy. Table 1 summarizes patient demographic and clinical information.

**Table 1.** Patient Demographics and Clinical Information

Mean age (SD, range), years	56.8 (10.7, 25–78)	
Sex (female:male)	30:15	
Brain Metastases	Radiation necrosis (*n* = 21)	Tumor (*n* = 31)
Primary tumor^a^		
Lung	9 (42.9%)	11 (35.5%)
Breast	4 (19.0%)	4 (12.9%)
Melanoma	3 (14.3%)	9 (29.0%)
Gastrointestinal		
Colorectal	1 (4.8%)	1 (3.2%)
Genitourinary		
Renal	1 (4.8%)	1 (3.2%)
Ovarian	1 (4.8%)	1 (3.2%)
Endometrial	0	1 (3.2%)
Testicular	2 (9.5%)	0
Head and neck		
Sarcoma	0	1 (3.2%)
Thymic	0	1 (3.2%)
Other		
Cardiac intima	0	1 (3.2%)
Stereotactic radiosurgery (SRS)		
Median fraction number (range)	1 (1–4)	1 (1–6)
Median radiation dose (range), Gy	24 (20–44)	24 (18–49)
Systemic therapy		
Chemotherapy	13 (61.9%)	16 (51.6%)
Immunotherapy	3 (14.3%)	4 (12.9%)
Both	5 (23.8%)	7 (22.6%)
None	0	4 (12.9%)
Median interval time from SRS to ASL-MRI (SD), days	331 (973.5)	273 (388.3)
Median interval time from ASL-MRI to histopathological confirmation (SD), days	18 (15.7)	8 (9.7)

^a^Percentages are relative to the total number of lesions within each column.

In the subgroup analysis of patients with both pre-SRS and post-SRS ASL-MRIs, 21 patients (mean age: 55.8, SD 9.6, range 33–70, 12 female) with a total of 23 suspected metastases (13 tumor, 10 RN) were evaluated. The primary cancers of origin were as follows: lung (*n* = 8), breast (*n* = 5), melanoma (*n* = 4), colorectal (*n* = 2), sarcoma (*n* = 1), ovarian (*n* = 1), thymic (*n* = 1), and cardiac (*n* = 1). Median interval time between pre-SRS ASL-MRI and initial SRS was 17 days (SD 9, range: 3–33) and between initial SRS and post-SRS ASL-MRI was 275 days (SD 302.5, range: 8–1058). Median interval time between pre-SRS and post-SRS ASL-MRIs was 301 days (SD 302.3, range: 21–1065). Median interval time between the post-SRS ASL-MRI and histopathological evaluation was 13 days (SD 11.9, range: 1–49). Lesions received a median dose of 24 Gy (SD 7.5, range 18–49) in a median fraction of 1 (SD 1.1, range 1–4). At the time of post-SRS ASL-MRI acquisition, 2 patients with 2 lesions had received both chemotherapy and immunotherapy, 14 patients with 15 lesions had received only chemotherapy, 5 patients with 5 lesions had received only immunotherapy, and 1 patient with 1 lesion had received neither chemotherapy nor immunotherapy.

### Tumor Versus Radiation Necrosis

**Figure 1.**
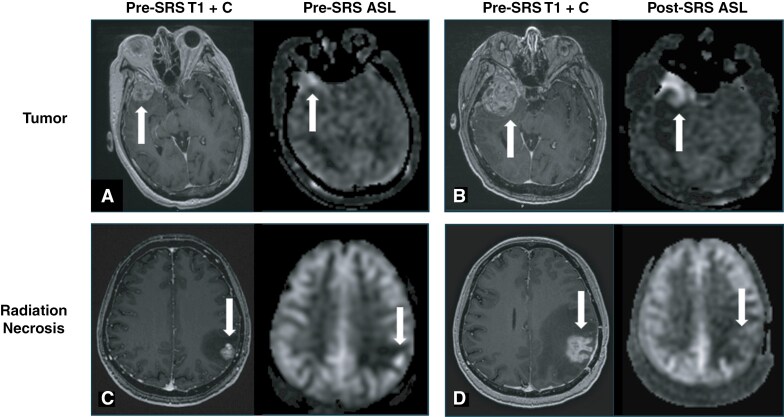
Representative examples of tumor and radiation necrosis. (A) Initial presenting contrast-enhancing tumor with elevated CBF in the right temporal lobe (white arrow) of a 65-year-old woman with metastatic thymic cancer. (B) Suspected recurrent tumor 8 days after SRS. MRI was obtained shortly after initial SRS to evaluate the patient’s new onset left facial droop following treatment. The contrast-enhancing mass is larger and demonstrates high CBF and markedly higher than on the pre-SRS image (white arrow). Histopathological evaluation after surgical resection revealed tumor. (C) Initial presenting contrast-enhancing tumor with elevated CBF in the left parietal lobe (white arrow) of a 42-year-old woman with metastatic breast cancer. (D) Suspected recurrent tumor 799 days after SRS. The contrast-enhancing mass is larger but demonstrates low CBF and lower than on the pre-SRS image (white arrow). Histopathological evaluation after surgical resection revealed radiation necrosis. Abbreviations: ASL = arterial spin labeling; T1 + C = T1-weighted with contrast; SRS = stereotactic radiosurgery.

Figure 1 shows representative examples of tumor and RN.

**Figure 2.**
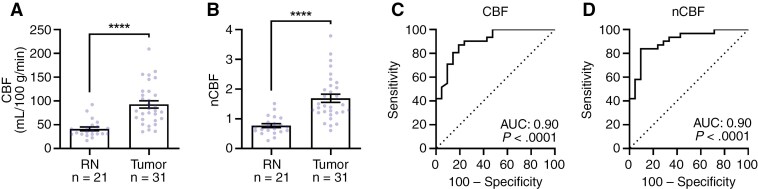
Absolute and normalized CBF differentiates between tumor and radiation necrosis. (A) Tumor exhibited significantly higher CBF compared to RN (tumor: mean ± SEM, 92.82 ± 7.70 mL/100 g/min; RN: mean ± SEM, 41.36 ± 4.02 mL/100 g/min; Mann–Whitney *U* test, *P* < .0001). (B) Tumor exhibited significantly higher normalized CBF compared to RN (tumor: mean ± SEM, 1.69 ± 0.14; RN: mean ± SEM, 0.77 ± 0.07; Mann–Whitney *U* test, *P* < .0001). (C) ROC for differentiating between tumor and RN using CBF. Performance was measured with an AUC of 0.90 (95% CI: 0.82–0.98, *P* < .0001). (D) ROC for differentiating between tumor and RN using normalized CBF. Performance was measured with an AUC of 0.90 (95% CI: 0.82–0.99, *P* < .0001). Data represented as mean ± SEM. All analyses were performed on *n* = 31 tumor lesions and *n* = 21 RN lesions. Blue circles represent individual lesions in A and B. Asterisks (****P < .0001) were used to denote levels of significance. Abbreviations: ASL = arterial spin labeling; CBF = cerebral blood flow; nCBF = normalized cerebral blood flow; RN = radiation necrosis.

Based on single timepoint post-SRS ASL-MRIs, tumor showed an increased mean absolute CBF of 92.82 mL/100 g/min (SEM 7.7, range 35.33–209.30) and normalized CBF (nCBF) of 1.69 (SEM 0.14, range 0.61–3.79), compared to RN which demonstrated a mean absolute CBF of 41.36 mL/100 g/min (SEM 4.02, range 16.67–92.33, *P* < .0001) and nCBF of 0.77 (SEM 0.07, range 0.27–1.51, *P* < .0001), respectively (Table 2; Figure 2A and B).

**Table 2.** CBF Metrics in Radiation Necrosis and Tumor Lesions

	Radiation necrosis	Tumor
CBF^a^^,^^b^^,^^c^	41.36 ± 4.02**** (16.67–92.33)	92.82 ± 7.70**** (35.33–209.30)
nCBF^a^^,^^b^	0.77 ± 0.07**** (0.27–1.51)	1.69 ± 0.14**** (0.61–3.79)
∆CBF^a^^,^^d^		
Absolute change^c^	−26.75 ± 13.43^*^ (−92.00 to 23.67)	21.56 ± 13.25^*^ (−104.0 to 74.00)
Percent change	−17.65 ± 15.19^*^ (−75.41 to 72.41)	50.55 ± 18.18^*^ (−49.92 to 180.00)
∆nCBF^a^^,^^d^		
Absolute change	−0.39 ± 0.19*** (−1.65 to 0.36)	0.49 ± 0.23*** (−1.02 to 2.67)
Percent change	−21.54 ± 9.40** (−73.28 to 30.79)	52.58 ± 20.34** (−31.45 to 237.70)

Abbreviations: CBF = cerebral blood flow; nCBF = normalized cerebral blood flow.

^a^Values reported as mean ± SEM (range).

^b^Based on a total of 52 lesions (radiation necrosis, *n* = 21; tumor, *n* = 31).

^c^Units: mL/100 g/min.

^d^Based on a total of 23 lesions (radiation necrosis, *n* = 10; tumor, *n* = 13).

*Denotes statistical significance between radiation necrosis and tumor with the Mann–Whitney *U* test.

^*^ *P* < .05, **P < .01, ***P < .001, ****P < .0001.

Performance (AUROC) of CBF and nCBF in differentiating tumor from RN was 0.90 for CBF (95% CI: 0.82–0.98, *P* < .0001) and 0.90 for nCBF (95% CI: 0.82–0.99, *P* < .0001) (Table 3; Figure 2C and D). Based on the Youden index, the optimal CBF threshold of 49.83 mL/100 g/min yielded a sensitivity of 87.1% and specificity of 81% to differentiate between RN and tumor. For nCBF, 1.13 yielded a sensitivity of 83.9% and specificity of 90.5%.

**Table 3.** ROC Curve Analyses for Differentiating Between Radiation Necrosis and Tumor Using CBF Metrics

	AUC (95% CI)	*P* value	Optimal threshold^a^	Sensitivity (%)	Specificity (%)
CBF^b^	0.90 (0.82, 0.98)	<.0001	49.83^c^	87.10	80.95
nCBF^b^	0.90 (0.82, 0.99)	<.0001	1.13	83.87	90.48
∆CBF^d^					
Absolute change	0.80 (0.61, 0.99)	.0156	29.17^c^	53.85	100.00
Percent change	0.81 (0.63, 0.98)	.0131	0.98	76.92	70.00
∆nCBF^d^					
Absolute change	0.89 (0.75, 1.00)	.0016	0.00	92.31	80.00
Percent change	0.88 (0.74, 1.00)	.0019	−0.10	92.31	80.00

Abbreviations: CBF = cerebral blood flow; nCBF = normalized cerebral blood flow.

^a^Based on Youden’s index.

^b^Based on a total of 52 lesions (radiation necrosis, *n* = 21; tumor, *n* = 31).

^c^Units: mL/100 g/min.

^d^Based on a subset of 23 lesions with both pre- and post-SRS ASL-MRIs (radiation necrosis, *n* = 10; tumor, *n* = 13).

### Effect of CBF Differences Between Pre-SRS and Post-SRS ASL-MRIs

**Figure 3.**
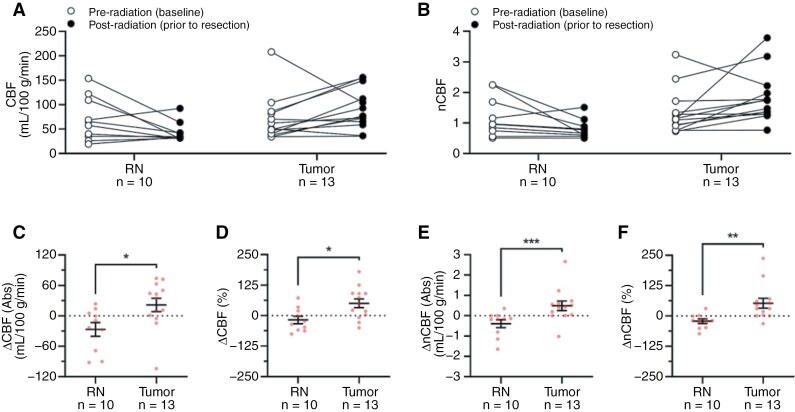
Absolute and normalized CBF obtained from pre- and post-radiation ASL-MRIs differentiate between tumor and radiation necrosis. (A, B) Visual graphs of CBF and nCBF in subgroup of patients with both pre-SRS and post-SRS ASL-MRIs. (C) Tumor exhibited significantly higher absolute ∆CBF (mean ± SEM, 21.56 ± 13.25 mL/100 g/min) compared with RN (mean ± SEM, −26.75 ± 13.43 mL/100 g/min; Mann–Whitney *U* test, *P* = .0147). (D) Tumor exhibited significantly higher percent ∆CBF (mean ± SEM, 50.55% ± 18.18%) compared with RN (mean ± SEM, −17.65% ± 15.19%; Mann–Whitney *U* test, *P* = .0121). (E) Tumor exhibited significantly higher absolute ∆nCBF (mean ± SEM, 0.49 ± 0.23) compared with RN (mean ± SEM, −0.39 ± 0.19; Mann–Whitney *U* test, *P* = .0009). (F) Tumor exhibited significantly higher percent ∆nCBF (mean ± SEM, 52.58% ± 20.34%) compared with RN (mean ± SEM, −21.54% ± 9.4%; Mann–Whitney *U* test, *P* = .0011). Data represented as mean ± SEM. All analyses were performed on *n* = 13 Tumor lesions and *n* = 10 RN lesions. White and black circles correspond to lesions with a paired pre- and post-radiation ASL-MRI in A and B. Red circles represent individual lesions in C–F. Asterisks (*P < .05, **P < .01, ***P < .001) were used to denote levels of significance. Abbreviations: Abs = absolute; ASL = arterial spin labeling; CBF = cerebral blood flow; nCBF = normalized cerebral blood flow; RN = radiation necrosis.

In the subgroup analysis consisting of lesions evaluated on both pre-SRS and post-SRS ASL-MRIs (13 tumor, 10 RN) (Figure 3), tumor exhibited significant increases in absolute and percent CBF differences before and after SRS (mean absolute ∆CBF: 21.56 mL/100 g/min, SEM 13.25, range −104 to 74; mean percent ∆CBF: 50.55%, SEM 18.18, range −49.92 to 180), in contrast to RN, which showed decreases in absolute and percent CBF differences (mean absolute ∆CBF: −26.75 mL/100 g/min, SEM 13.43, range −92 to 23.67, *P =* .0147; mean percent ∆CBF: −17.65%, SEM 15.19, range −75.41 to 72.41, *P* = .0121) (Table 2; Figure 3C and D). AUROC of absolute ∆CBF was 0.80 (95% CI, 0.61–0.99, *P* = .0156), with a sensitivity of 53.9% and specificity of 100% when using the optimal threshold of 29.17 mL/100 g/min (Table 3). For percent ∆CBF, AUROC was 0.81 (95% CI, 0.63–0.98, *P* = .0131), with a sensitivity of 76.9% and specificity of 70% based on the threshold of 0.98% (Table 3).

Similarly, when evaluating nCBF, tumor showed significant increases in absolute and percent differences before and after SRS (mean absolute ∆nCBF: 0.49, SEM 0.23, range −1.02 to 2.67; mean percent ∆nCBF: 52.58%, SEM 20.34, range −31.45 to 237.70), compared to RN, which showed decreases in absolute and percent differences (mean absolute ∆nCBF: −0.39, SEM 0.19, range −1.65 to 0.36, *P *= .0009; mean percent ∆nCBF: −21.54%, SEM 9.4, range −73.28 to 30.79, *P *= .0011) (Table 2; Figure 3E and F). AUROC of absolute ∆nCBF was 0.89 (95% CI, 0.75–1.00, *P *= .0016), with a sensitivity of 92.3% and specificity of 80% based on the optimal threshold of 0 (Table 3). For percent ∆nCBF, AUROC was 0.88 (95% CI, 0.74–1.00, *P* = .0019), with a sensitivity of 92.3% and specificity of 80% based on the threshold of −0.10% (Table 3).

Of the 13 tumor lesions, 3 lesions showed negative ∆CBF (mean absolute ∆CBF: −41.22 mL/100 g/min, range: −5.33 to −104; mean percent ∆CBF: −28.6%, range: −7.58 to −49.92), and one showed negative ∆nCBF (absolute ∆nCBF: −1.02 mL/100 g/min; percent ∆nCBF: −31.45%) between both pre-SRS and post-SRS timepoints. Evaluation of the single timepoint post-SRS CBF and nCBF of these lesions revealed mean values of 68.55 and 1.75 mL/100 g/min, respectively.

Of 10 RN lesions, 3 lesions showed positive ∆CBF (mean absolute ∆CBF: 14.27 mL/100 g/min, range: 5.13–23.67; mean percent ∆CBF: 42.21%, range: 19.74–72.41), and 2 lesions showed positive ∆nCBF (mean absolute ∆nCBF: 0.18 mL/100 g/min, range: 0.01–0.36; mean percent ∆nCBF: 16.34%, range: 1.88–30.79). Evaluation of the single timepoint post-SRS CBF and nCBF of these lesions revealed mean values of 52.27 and 0.86 mL/ 100 g/min, respectively.

Supplementary Figure 1 shows representative examples of false negative and false positive lesions.

### Mixed Lesions

In supplementary analyses, 21 mixed lesions in 18 patients were compared with RN and tumor (same groups as in the primary analyses) and showed CBF values in between RN and tumor across all CBF metrics (Supplementary Tables 1 and 2; Supplementary Figure 2). Tumor exhibited significantly higher CBF (RN: mean ± SEM, 41.36 ± 4.02 mL/100 g/min; mixed lesions: 64.71 ± 6.10 mL/100 g/min; tumor: 92.82 ± 7.70 mL/100 g/min; *P* < .0001) and higher nCBF (RN: mean ± SEM, 0.77 ± 0.07; mixed: 1.23 ± 0.14; tumor: 1.69 ± 0.14; Tukey’s multiple comparisons tests via one-way ANOVA, *P* < .0001) compared to RN and mixed lesions.

### Effect of Time After Radiotherapy

In RN lesions (*n* = 21), the median interval time between initial SRS and post-SRS ASL-MRI was 331 days (SD 973.5, range: 161–3547). When stratifying RN lesions based on time after receiving radiotherapy (5 months–2 years and >2 years), no significant differences were observed with either single timepoint post-SRS CBF or nCBF (*P *= .62 and *P *= .29, respectively) (Supplementary Table 3). Simple linear regression analysis also revealed no significant relationship with either CBF or nCBF and time after radiotherapy (*R*^2^ = 0.02, *P *= .54 and *R*^2^ = 0.00, *P *= .82, respectively) (Supplementary Table 4).

## Discussion

Our results demonstrate that the use of ASL-derived CBF performed well to differentiate between tumor and RN in BM previously treated with SRS. Absolute and normalized CBF metrics performed similarly to achieve this task. While knowledge of baseline pre-SRS and post-SRS CBF (∆CBF) also performed well to differentiate between tumor and RN in this patient population, performance was comparable and not superior to that achieved by knowledge of only post-SRS CBF obtained at a single timepoint. Finally, in RN, we found no significant relationship between CBF and time after radiotherapy. Overall, our results demonstrate ASL-MRI’s value in differentiating tumor from RN in various types of BM following SRS.

To date, few studies have reported on ASL-MRI’s ability to distinguish between tumor and RN in post-SRS BM, including Kuo et al. who reported an AUROC of 0.71 in 26 patients^5^ and Lai et al. who reported an accuracy of 93%, sensitivity of 83%, and specificity of 100% in 14 patients.^6^ These results correlate well with studies using DSC, in which the use of relative CBV or fractional tumor burden was comparable in differentiating between tumor and RN in this patient population.^5,11–13,17,18^ Other studies of ASL-MRI in post-SRS BM have evaluated its utility in monitoring blood flow response to SRS and in predicting treatment outcomes.^13,16,20,21^ Our study expands on the results of these studies with a larger sample size, using histopathological diagnosis as the ground truth. The latter is particularly important because clinical criteria can be more vulnerable to subjective assessment.^3,5,6,9,14–18^ In addition, our use of histopathological diagnosis was strict as we included only lesions with definitive diagnoses of tumor or RN in our primary analyses. However, in practice, mixed lesions containing a combination of tumor and RN features can occur^24–26^ and can be difficult to tease out on imaging. Since we did not know the proportion of tumor and RN in each sample of a mixed lesion, we excluded these lesions from the primary analyses, in large part because varying proportions of tumor and RN can affect CBF measurements. Out of interest, we did compare CBF measurements of mixed lesions relative to tumor and RN lesions in supplementary analyses, and as expected, CBF values for mixed lesions were in between CBF values of tumor and RN. A future study is needed to determine if ASL-MRI can predict tumor proportion within mixed lesions. Nonetheless, our results provide further validation of ASL-MRI’s ability to differentiate between tumor and RN in the post-SRS BM setting.

It is reasonable to infer that knowledge of pre-SRS tumor perfusion may help to guide interpretation of post-SRS MRIs.^10,16,19,20,22^ Lambert and Holmes^16^ found that at baseline, approximately 40% of BM have ASL-derived blood flow lesser or equal to CBF of healthy contralateral cortex, suggesting that possible recurrence following SRS may not be characterized by elevated blood flow. Therefore, ASL-MRI may not be sensitive enough to identify tumor recurrence in non-hypervascular lesions and may lead to false negative diagnoses. Our results show that the added knowledge of pre-SRS CBF (assessed by ∆CBF between pre-SRS and post-SRS timepoints) performed well to differentiate between tumor and RN. The performance was comparable but not superior to CBF obtained from a single timepoint post-SRS. AUROC for single and dual timepoints ranged from 0.80 to 0.90. This is likely because RN lesions showed significantly decreased CBF on post-SRS ASL-MRI in both non-hypervascular and hypervascular lesions, such that between pre-SRS and post-SRS ASL-MRI timepoints, tumor was typically associated with a positive difference in ∆CBF while RN was associated with a negative difference in ∆CBF. This may also help to explain why we found no significant relationship between CBF and time after radiotherapy, as RN lesions tend to consistently show low blood flow across time.

This trend did not hold true for a minority of lesions in either condition, and there were minor discrepancies between ∆CBF and ∆nCBF in false positive and false negative lesions. While we found that there was overall similar performance of absolute and normalized CBF to differentiate between tumor and RN, normalized ∆CBF slightly outperformed absolute ∆CBF. This may be because nCBF helps to mitigate the widespread interpatient variation that can be found with absolute CBF, which can be modulated by factors such as physiologic state, breathing, and caffeine intake. By accounting for global variation between individuals and standardizing the analyses, nCBF has been shown to have greater sensitivity compared with absolute CBF, even when the nCBF differences and signal-to-noise ratio are low between patients and controls.^27^ Nonetheless, the ease of obtaining both measurements increases the generalizability and adoption of ASL-MRI. Further investigation of the false negative lesions (tumor lesions that showed negative ∆CBF or ∆nCBF) revealed some plausible explanations for lower blood flow on post-SRS ASL-MRI: development of large hematoma at the site of the irradiated lesion, which can obscure ASL signal (eg, due to mass effect and compression) found in one patient, as RN is typically associated with lower blood flow than tumor. In the 2 other patients, it is possible that tumor may proliferate without the need for increased angiogenesis, such as through the upregulation of non-angiogenic, invasion-focused transcription factors.^28^ Nonetheless, it is worthwhile to note that for the majority of these lesions, single timepoint post-SRS ASL-MRI showed higher CBF and nCBF as expected for tumor. Further investigation of the false positive lesions (RN lesions that showed positive ∆CBF or ∆nCBF) suggested a possible explanation for higher blood flow: the ability of post-SRS inflammation and edema to induce increased blood flow in the RN microenvironment.^21^ In the majority of these lesions, however, the single timepoint post-SRS ASL-MRI showed lower CBF and nCBF as expected for RN. For these reasons, an uptrending ∆CBF or ∆nCBF is not always associated with recurrent tumor, and a downtrending ∆CBF or ∆nCBF is not always associated with RN.

In post-SRS BM, ASL-MRI is an important tool and may provide advantages over conventional contrast-enhanced MRI and DSC-MRI. For the diagnosis of tumor and RN, studies have shown poor performance of contrast-enhanced MRI,^5–7^ with reported AUROC of 0.64^5^ and accuracy of 46%.^6^ Moreover, while results from ASL and DSC often correlate well, ASL can provide more useful information in cases of hemorrhagic BMs (particularly since BM can develop hemorrhage after SRS) and BMs located near large vessels or bone or scalp. These cases can obscure DSC imaging and postprocessing due to susceptibility. Kerkhoff et al. reported that 15% (26/168) of cases could not be evaluated due to such susceptibility artifact.^10^ Nonetheless, when evaluating post-SRS lesions on surveillance imaging, the choice of either ASL or DSC is dependent on the institution (eg, related to the preferred perfusion protocol and postprocessing software or method). However, when assessing lesions that are hemorrhagic or near air or bone structures, all of which can obscure the signal on DSC, we recommend using ASL for improved interpretation. Based on our results, one can be more confident that a post-SRS contrast-enhancing lesion ≥10 mm represents RN if absolute maximum CBF < 50 mL/100 g/min and normalized maximum CBF < 1.13 on ASL-MRI.

Our study has important limitations. First, the relatively small sample size consisting of only retrospective data from a single institution limits the generalizability of our results. In addition, although we included a variety of tumor histology in our analysis, the predominant primary cancers of origin included were lung and breast with very few originating from gastrointestinal or genitourinary locations. Due to this discrepancy, a larger (ideally multicenter) study including more cancer types is needed to determine ASL-MRI’s ability to accurately distinguish between tumor and RN across various histology. Similarly, a larger and more equal distribution of hypervascular and non-hypervascular pre-SRS lesions could potentially reveal a significant effect on ASL-MRI’s performance that our current study’s sample size was too small to capture. We also acknowledge a selection bias as only lesions that underwent surgical resection were included in the study. However, only histopathologic proven lesions were included because we wanted definitive evidence of RN and tumor as diagnoses made based on follow-up imaging can be subjective. As previously mentioned, mixed lesions can occur in practice. We did not include mixed lesions in the primary analyses because we did not know the proportion of tumor and RN in each sample. Ground truth diagnoses were also obtained exclusively from the histopathology reports. It would have been ideal for a neuropathologist to reevaluate all samples in the study for the relative presence of tumor and RN rather than relying on a description or lack of description of these entities in the reports. However, it would be challenging to analyze histopathology of all samples collected over a timespan of nearly 25 years, particularly of the older samples (not all of which are available), in a consistent and reliable way. In future studies, it will be important to determine the proportion of tumor and RN in surgical samples, as that may affect CBF values. In addition, the inclusion of only surgically resected lesions minimizes the possibility of insufficient sampling and subsequent false negatives that may occur in lesions that were sampled via biopsy only.^29,30^

In conclusion, our study demonstrates that ASL-MRI-derived CBF values exhibit great promise in differentiating between viable tumor and RN in previously irradiated BM. The ability to effectively accomplish this task without the need for knowledge of pre-radiation blood flow status and independent of time after radiotherapy provides for great promise in widespread adoption and implementation at institutions. Features of ASL-MRI including noncontrast acquisition, rapid and automated image postprocessing, and less propensity for uninterpretable images related to susceptibility artifacts seen with DSC make it an ideal imaging option in this patient population. Despite such promising results, further clinical and histologic validation in larger prospective studies is needed to determine its benefits in patients with various cancer types.

## Supplementary Material

vdaf091_suppl_Supplementary_Figure_S1

vdaf091_suppl_Supplementary_Figure_S2

vdaf091_suppl_Supplementary_Materials

## Data Availability

Data shall be made available upon reasonable request. For access, please contact corresponding author, Michael Iv, at miv@stanford.edu.
