# Supplementary figures and images for: Arterial spin labeling perfusion MRI differentiates between radiation necrosis and tumor in brain metastases treated with stereotactic radiosurgery
Source: Neurooncol Adv. 2025 May 7;7(1):vdaf091. doi: 10.1093/noajnl/vdaf091 (PMC12202033; doi:10.1093/noajnl/vdaf091)

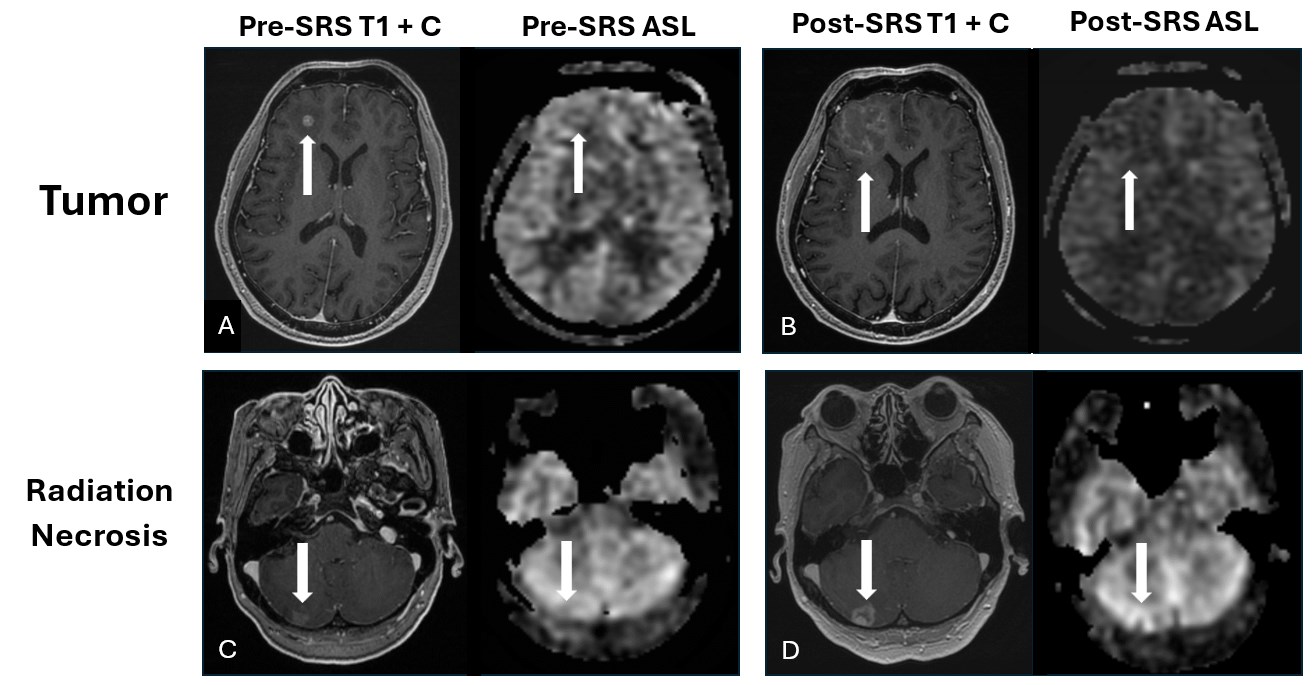

Supplement: vdaf091_suppl_Supplementary_Figure_S1 [file vdaf091_suppl_supplementary_figure_s1.jpeg]

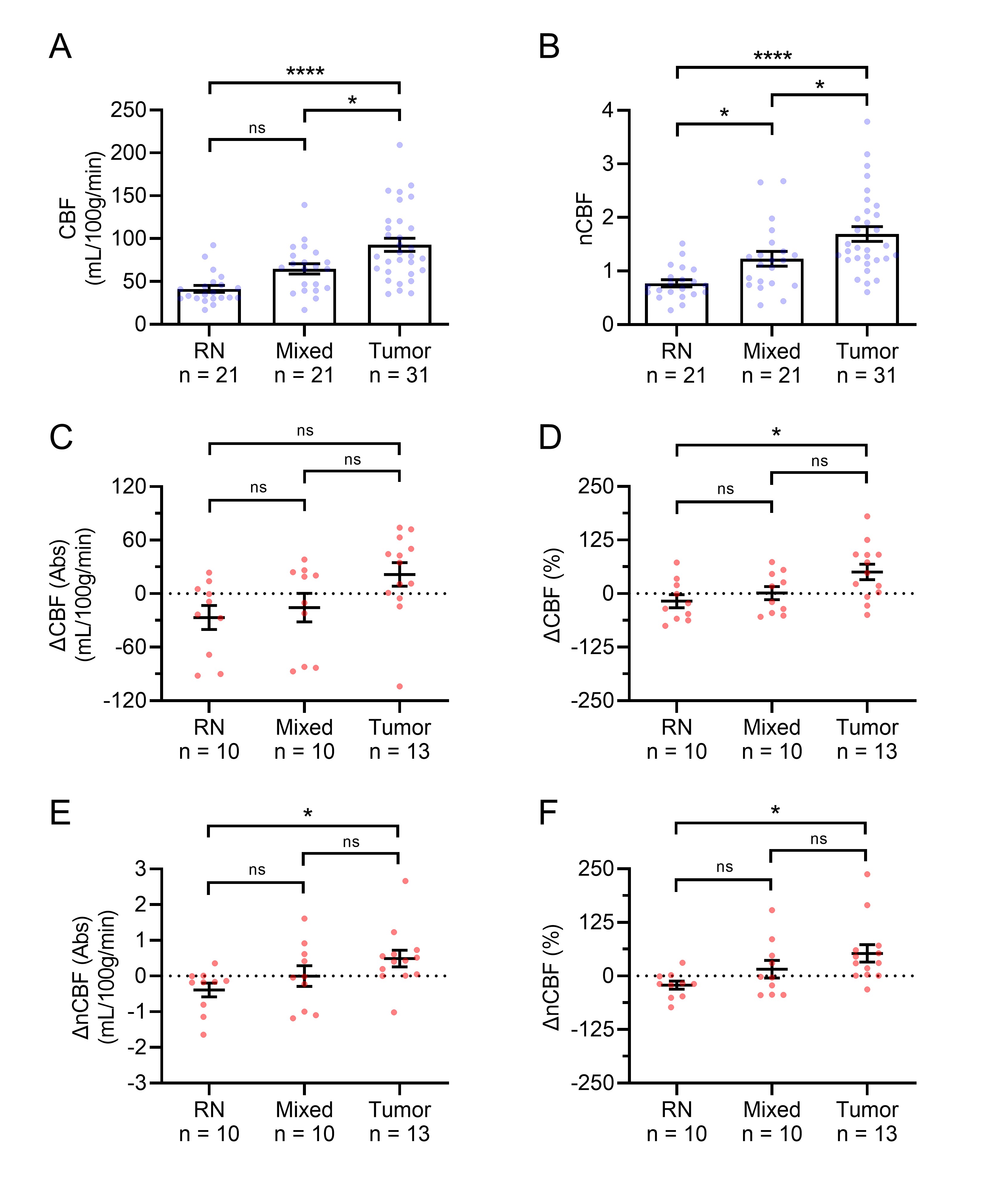

Supplement: vdaf091_suppl_Supplementary_Figure_S2 [file vdaf091_suppl_supplementary_figure_s2.jpeg]
