# Supplementary material for: Arterial spin labeling perfusion MRI differentiates between radiation necrosis and tumor in brain metastases treated with stereotactic radiosurgery
Source: Neurooncol Adv. 2025 May 7;7(1):vdaf091. doi: 10.1093/noajnl/vdaf091 (PMC12202033; doi:10.1093/noajnl/vdaf091)
Supplement: vdaf091_suppl_Supplementary_Materials [file vdaf091_suppl_supplementary_materials.docx]

**
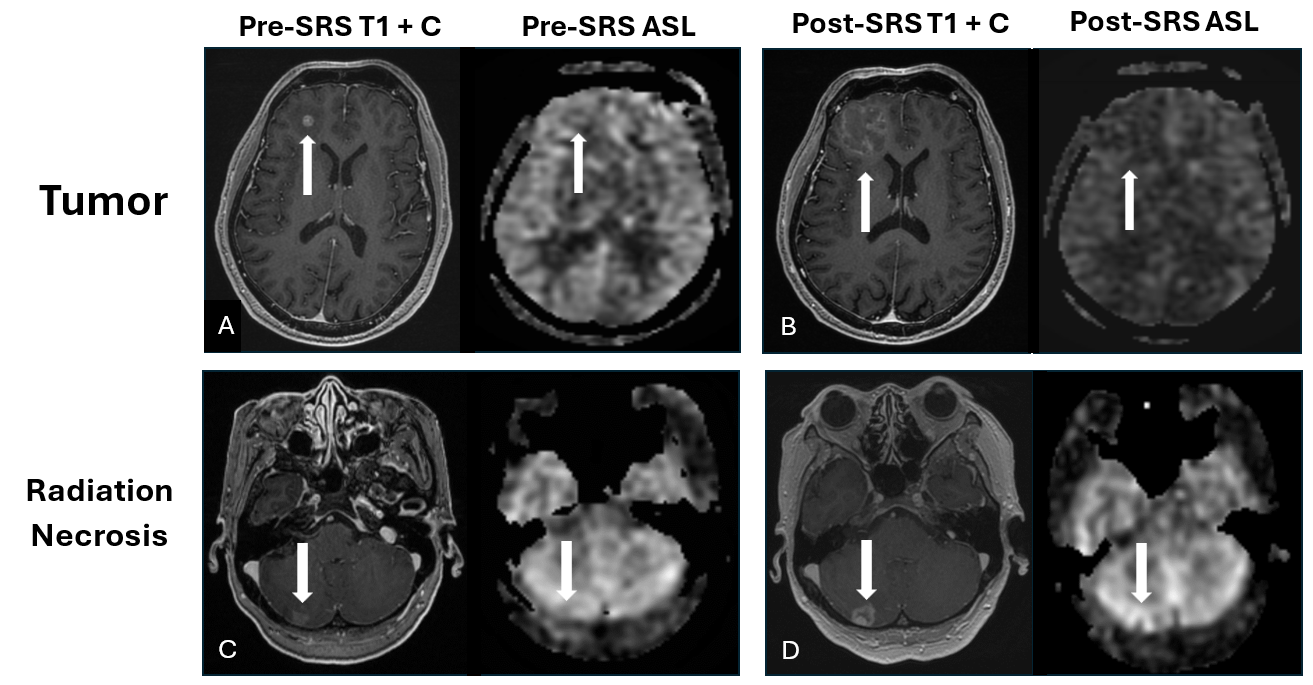
**

**Supplementary Figure 1.** Representative examples of false negative (tumor that showed negative ∆CBF, defined as post-SRS CBF – pre-SRS CBF) and false positive (RN that showed positive ∆CBF) lesions.

(A) Initial presenting contrast-enhancing tumor with elevated CBF in the right frontal lobe (white arrow) of a 55-year-old man with metastatic lung cancer.

(B) Suspected recurrent tumor 273 days after SRS. The contrast-enhancing mass is larger but demonstrates lower CBF than on the pre-SRS image (white arrow). Histopathological evaluation after surgical resection revealed tumor.

(C) Initial presenting contrast-enhancing tumor with mildly elevated CBF in the right cerebellum (white arrow) of a 59-year-old woman with metastatic lung cancer.

(D) Suspected recurrent tumor 300 days after SRS. The contrast-enhancing mass is larger and demonstrates slightly higher CBF than on the pre-SRS image (white arrow). Histopathological evaluation after surgical resection revealed radiation necrosis.

Abbreviations: ASL – arterial spin labeling; T1 + C – T1-weighted with contrast; SRS – stereotactic radiosurgery


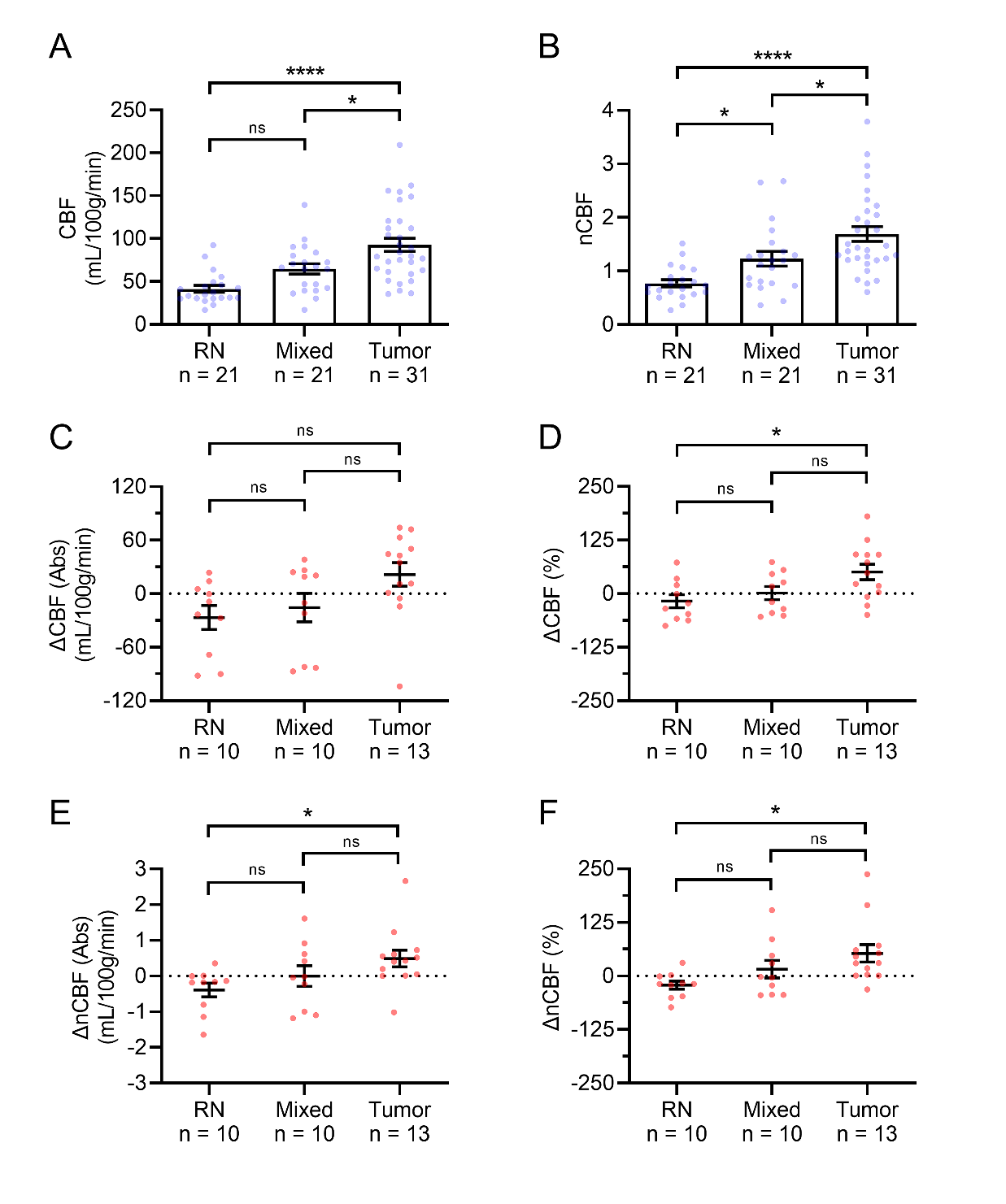


**Supplementary Figure 2.** Absolute, normalized, and change in CBF obtained from pre- and post-radiation ASL-MRIs in radiation necrosis, mixed, and tumor lesions.

(A) Tumor exhibited significantly higher CBF compared to RN and mixed lesions (RN: mean ± SEM, 41.36 ± 4.02 mL/100 g/min; mixed: mean ± SEM, 64.71 ± 6.10 mL/100 g/min; tumor: mean ± SEM, 92.82 ± 7.70 mL/100 g/min; Tukey’s multiple comparisons tests via one-way ANOVA, *P* < .0001).

(B) Tumor exhibited significantly higher normalized CBF compared to RN and mixed lesions (RN: mean ± SEM, 0.77 ± 0.07; mixed: mean ± SEM, 1.23 ± 0.14; tumor: mean ± SEM, 1.69 ± 0.14; Tukey’s multiple comparisons tests via one-way ANOVA, *P* < .0001).

(C) No significant difference in absolute ∆CBF between RN, mixed lesions, and tumor (RN: mean ± SEM, -26.75 ± 13.43 mL/100 g/min; mixed: mean ± SEM, -15.63 ± 15.93; tumor: mean ± SEM, 21.56 ± 13.25 mL/100 g/min; Tukey’s multiple comparisons tests via one-way ANOVA, *P* = .047).

(D) Tumor exhibited significantly higher percent ∆CBF compared with RN (RN: mean ± SEM, -17.65 ± 15.19%; mixed: mean ± SEM, 1.25 ± 15.22; tumor: mean ± SEM, mean ± SEM, 50.55 ± 18.18%; Tukey’s multiple comparisons tests via one-way ANOVA, *P* = .017).

(E) Tumor exhibited significantly higher absolute ∆nCBF compared with RN (RN: mean ± SEM, -0.39 ± 0.19%; mixed: mean ± SEM, 0.00 ± 0.29%; tumor: mean ± SEM, mean ± SEM, 0.49 ± 0.23%; Tukey’s multiple comparisons tests via one-way ANOVA, *P* = .045).

(F) Tumor exhibited significantly higher percent ∆nCBF compared with RN (RN: mean ± SEM, -21.54 ± 9.40%; mixed: mean ± SEM, 15.70 ± 20.56%; tumor: mean ± SEM, mean ± SEM, 52.58 ± 20.34%; Tukey’s multiple comparisons tests via one-way ANOVA, *P* = .024).

**P* < .05, *****P* < .0001

Abbreviations: CBF – cerebral blood flow; nCBF – normalized cerebral blood flow; RN – radiation necrosis

**Supplementary Table 1.** Patient demographics and clinical information for mixed lesions.

| Mean age (SD, range), years | 57.1 (10.2, 41-73) |
| --- | --- |
| Sex (female:male) | 12:6 |
|  |  |
| Brain Metastases | (n = 21) |
| Primary tumor^a^ |  |
| Lung | 6 (28.6%) |
| Breast | 7 (33.3%) |
| Melanoma | 3 (14.3%) |
| Gastrointestinal |  |
| Duodenal | 1 (4.8%) |
| Colorectal | 2 (9.5%) |
| Genitourinary |  |
| Renal | 1 (4.8%) |
| Head & neck |  |
| Frontal sinus | 1 (4.8%) |
|  |  |
| Stereotactic radiosurgery (SRS) |  |
| Median fraction number (range) | 2 (1 – 6) |
| Median radiation dose (range), Gy | 24 (18 – 54) |
|  |  |
| Systemic therapy |  |
| Chemotherapy | 7 (33.3%) |
| Immunotherapy | 4 (19.0%) |
| Both | 9 (42.9%) |
| None | 1 (4.8.%) |
|  |  |
| Median interval time from SRS to ASL-MRI (SD), days | 297 (386.2) |
| Median interval time from ASL-MRI to histopathological confirmation (SD), days | 9 (7.7) |

^a^Percentages are relative to the total number of lesions within each column.

**Supplementary Table 2**: CBF metrics in radiation necrosis, mixed lesions, and tumor lesions.

|  | Radiation Necrosis | Mixed | Tumor |
| --- | --- | --- | --- |
| CBF^a,b,c^ | 41.36 ± 4.02^####^  (16.67 – 92.33) | 64.71 ± 6.10^&^  (16.87 – 139.30) | 92.82 ± 7.70^####,&^  (35.33 – 209.30) |
| nCBF^a,b^ | 0.77 ± 0.07*^,####^  (0.27 – 1.51) | 1.23 ± 0.14*^,&^  (0.36 – 2.68) | 1.69 ± 0.14^####,&^  (0.61 – 3.79) |
|  |  |  |  |
| ∆CBF^a,d^ |  |  |  |
| Absolute change^c^ | -26.75 ± 13.43  (-92.00 – 23.67) | -15.63 ± 15.93  (-87.00 – 38.33) | 21.56 ± 13.25  (-104.0, 74.00) |
| Percent change | -17.65 ± 15.19^#^  (-75.41 – 72.41) | 1.25 ± 15.22  (-54.01 – 73.72) | 50.55 ± 18.18^#^  (-49.92, 180.00) |
|  |  |  |  |
| ∆nCBF^a,c^ |  |  |  |
| Absolute change | -0.39 ± 0.19^#^  (-1.65 – 0.36) | 0.00 ± 0.29  (-1.18 – 1.61) | 0.49 ± 0.23^#^  (-1.02 – 2.67) |
| Percent change | -21.54 ± 9.40^#^  (-73.28 – 30.79) | 15.70 ± 20.56  (-44.84 – 153.80) | 52.58 ± 20.34^#^  (-31.45 – 237.70) |
|  |  |  |  |

^a^Values reported as mean ± SEM (range).

^b^Based on a total of 73 lesions (Radiation Necrosis, n = 21; Mixed = 21, Tumor, n = 31).

^c^Units: mL/100 g/min

^d^Based on a total of 33 lesions (Radiation Necrosis, n = 10; Mixed = 10, Tumor, n = 13).

* denotes statistical significance, based on multiplicity-adjusted *P* values, between Radiation Necrosis and Mixed with Tukey’s multiple comparisons tests via one-way ANOVA analysis. **P* < .05, *****P* < .0001.

# denotes statistical significance, based on multiplicity-adjusted *P* values, between Radiation Necrosis and Tumor with Tukey’s multiple comparisons tests via one-way ANOVA analysis. ^#^*P* < .05.

& denotes statistical significance, based on multiplicity-adjusted *P* values, between Mixed and Tumor with Tukey’s multiple comparisons tests via one-way ANOVA analysis. ^&^*P* < .05.

Abbreviations: CBF – cerebral blood flow; nCBF – normalized cerebral blood flow

**Supplementary Table 3.** Cerebral blood flow values of radiation necrosis lesions derived from single timepoint post-SRS ASL-MRI, stratified by the number of years following radiotherapy.

|  | 5 mo – 2 y  since radiation  (n = 14) | >2 y  since radiation  (n = 7) | *P* value^c^ |
| --- | --- | --- | --- |
| CBF^a,b^ | 39.74  (29.72, 49.77) | 44.00  (23.60, 64.40) | 0.62 |
| nCBF^a^ | 0.74  (0.57, 0.90) | 0.87  (0.57, 1.17) | 0.29 |
|  |  |  |  |

^a^Values reported as mean and 95% CI.

^b^Units: mL/100 g/min.

^c^Mann-Whitney *U* test.

Abbreviations: ASL – arterial spin labeling; CBF – cerebral blood flow; nCBF – normalized cerebral blood flow; RN – radiation necrosis; SRS – stereotactic radiosurgery

**Supplementary Table 4.** Best-fit values generated from two simple linear regression models using time since radiation as an independent variable, with either CBF or nCBF as dependent variables.

| Dependent Variable | Slope^a^ | Y intercept^a^ | Goodness of Fit, R^2^ | *P* value |
| --- | --- | --- | --- | --- |
| CBF | -0.99^b^  (-4.31, 2.33) | 43.29^c^  (32.09, 54.49) | 0.02 | 0.54 |
| nCBF | 0.01  (-0.06, 0.05) | 0.79  (0.61, 0.97) | 0.00 | 0.82 |
|  |  |  |  |  |

^a^Values reported as the best-fit value with 95% CI.

^b^Units: mL/100 g/min, per year since radiation.

^c^Units: mL/100 g/min.

Abbreviations: CBF – cerebral blood flow; nCBF – normalized cerebral blood flow
